# Supplementary material for: Striking circadian neuron diversity and cycling of Drosophila alternative splicing
Source: eLife. 2018 Jun 4;7:e35618. doi: 10.7554/eLife.35618 (PMC6025963; doi:10.7554/eLife.35618)
Supplement: Supplementary file 1. — The distribution of splice site type in the AS junctions (including reverse strand) detected in each neuron group/sample that are within a gene. [file elife-35618-supp1.docx]

**Supplementary File 1.** The distribution of splice site type (including reverse strand) in the AS junctions detected in each neuron group/sample.

| Neuron group/sample | GT/AG | CT/AC | GC/AG | CT/GC | AT/AC | GT/AT | Non-canonical |
| --- | --- | --- | --- | --- | --- | --- | --- |
| DN1 | 7,650 | 7,888 | 40 | 42 | 4 | 4 | 0 |
| LNd | 7,247 | 7,470 | 37 | 26 | 4 | 2 | 0 |
| LNv | 7,367 | 7,704 | 40 | 32 | 0 | 2 | 0 |
| TH | 6,374 | 6,646 | 37 | 26 | 2 | 4 | 0 |
| Head | 7,114 | 7,023 | 57 | 48 | 0 | 2 | 2 |

The distribution of splice site type in the AS junctions (including reverse strand) detected in each neuron group/sample that are within a gene.

| Neuron group/sample | GT/AG | CT/AC | GC/AG | CT/GC | AT/AC | GT/AT | Non-canonical |
| --- | --- | --- | --- | --- | --- | --- | --- |
| DN1 | 3,637 | 3,429 | 25 | 21 | 0 | 2 | 0 |
| LNd | 3,941 | 3,708 | 22 | 15 | 0 | 0 | 0 |
| LNv | 3,651 | 3,624 | 23 | 14 | 0 | 2 | 0 |
| TH | 3,233 | 3,185 | 20 | 14 | 0 | 2 | 0 |
| Whole head | 6,976 | 6,884 | 57 | 45 | 0 | 0 | 0 |
