# Supplementary material for: Striking circadian neuron diversity and cycling of Drosophila alternative splicing
Source: eLife. 2018 Jun 4;7:e35618. doi: 10.7554/eLife.35618 (PMC6025963; doi:10.7554/eLife.35618)
Supplement: Supplementary file 2. [file elife-35618-supp2.docx]

**Supplementary File 2.** Gene ontology analyses of those transcripts that present neuron subgroup specific novel ALT junctions that are not found in whole head samples for each neuron subpopulation.

| **Neuron subgroup** | **GO category** | **Genes** |
| --- | --- | --- |
| LNv | Neurotransmitter secretion | *CASK, cac, Syt1, Syn, Snap25, unc-13, Chc, Syx16, Arf69F, Rab3-GEF, cm, stnB, Sytalpha* |
|  | Negative regulation of translation | *sqd, bru1, Rbfox1, bru2, Sxl, smg, pum* |
| LNd | Acetylcholine-activated cation-selective channel activity | *nAChRbeta2, nAChRbeta1, nAChRalpha4, nAChRalpha5, nACHRalpha6* |
|  | Synaptic target recognition | *Sema1a*, *galectin*, *tutl*, *hig*, *ab*, *Ten-a* |
| DN1 | Potassium ion transporters | *Shab*, *Sh*, *nrv3*, *ATPalpha*, *slo*, *Irk2* and *Hk* |
|  | Locomotor rhythms | *cry*, *Mef2*, *sgg*, *Mid1*, *Ac3*, *dnc*, *gw*, *NPF* |
| TH | chemical synaptic transmission | *Sap47, Shab, cac, Syt1, dnc, pAbp, pum, CaMKII, Ace, ChAT, Vmat* |
