# Supplementary material for: Striking circadian neuron diversity and cycling of Drosophila alternative splicing
Source: eLife. 2018 Jun 4;7:e35618. doi: 10.7554/eLife.35618 (PMC6025963; doi:10.7554/eLife.35618)
Supplement: Supplementary file 3. [file elife-35618-supp3.docx]

**Supplementary File 3.** Gene ontology analyses of those transcripts that present neuron subgroup specific cycling AS structures.

| **Neuron subgroup** | **GO category** | **Genes** |
| --- | --- | --- |
| LNv | Locomotor behavior | *CASK*, *Nrx-1* and *unc-104* |
|  | Male courtship behavior | *CASK*, *syn*, *fne*, *Gr43a*, *pros* |
| LNd | Locomotor behavior | *dnc*, *gw*, *sgg*, *Fer2LCH* |
|  | Olfactory learning | *PkaR1*, *klg*, *dnc*, *dikar*, *sgg* |
| DN1 | Locomotor behavior | *unc80*, *dlg1*, *dnc*, *PdfR*, *unc79* |
|  | mRNA binding proteins | *B52*, *HnRNP-K*, *Syp*, *heph*, *shep*, *ps*, *cop*, *bru3*, *Sxl*, *Rox8*, *Rb97b*, CG7879, *rump* |
