# Supplementary material for: Striking circadian neuron diversity and cycling of Drosophila alternative splicing
Source: eLife. 2018 Jun 4;7:e35618. doi: 10.7554/eLife.35618 (PMC6025963; doi:10.7554/eLife.35618)
Supplement: Supplementary file 4. [file elife-35618-supp4.docx]

**Supplementary File 4**. Mapping statistics of the neuronal RNA-seq data to the Drosophila genome (dm3)

| Neuronal sample (collapsed from six time points data for each independent day, respectively, for hand-dissected neurons) | # of total reads | # of uniquely mapped reads |
| --- | --- | --- |
| DN1 replicate A | 185,846,361 | 128,170,121 (68.97%) |
| DN1 replicate B | 194,725,264 | 122,606,031 (62.96%) |
| LNd replicate A | 175,540,369 | 104,199,531 (59.36%) |
| LNd replicate B | 195,124,649 | 168,030,537 (86.11%) |
| LNv replicate A | 174,841,442 | 134,755,240 (77.07%) |
| LNv replicate B | 186,636,889 | 149,057,797 (79.87%) |
| TH replicate A | 171,527,204 | 93,449,460 (54.48%) |
| TH replicate B | 168,619,569 | 156,029,405 (92.53%) |
| Head | 205,576,030 | 176,458,018 (85.84%) |
