# Supplementary material for: Striking circadian neuron diversity and cycling of Drosophila alternative splicing
Source: eLife. 2018 Jun 4;7:e35618. doi: 10.7554/eLife.35618 (PMC6025963; doi:10.7554/eLife.35618)
Supplement: Supplementary file 5. [file elife-35618-supp5.docx]

**Supplementary File 5.**  Commands for STAR mapping.

**First round**:

Generating genome index:

STAR --runThreadN 3 --runMode genomeGenerate --genomeDir genome_index_STAR_r1 --genomeFastaFiles dm3.fa --sjdbGTFfile dm3_genes.gtf --sjdbOverhang 50

Mapping:

STAR --runThreadN 3 --genomeDir genome_index_STAR_r1 --outFileNamePrefix DN1_A --readFilesIn DN1_A_R01.fastq DN1_A_R02.fastq --outSJfilterReads Unique

**Second round**:

Re-generating genome index:

STAR --runThreadN 3 --runMode genomeGenerate --genomeDir genome_index_STAR_r2 --genomeFastaFiles dm3.fa --sjdbFileChrStartEnd combined_SJ_out_tab_unannotated_for_2nd_pass_genome_generation.txt --sjdbGTFfile dm3_genes.gtf --sjdbOverhang 50

- *combined_SJ_out_tab_unannotated_for_2nd_pass_genome_generation.txt is an output file from running JUM. For details, see JUM manual at: https://github.com/qqwang-berkeley/JUM*

Mapping:

STAR --runThreadN 3 --genomeDir genome_index_STAR_r2 --outFileNamePrefix ctrl_2nd --readFilesIn ctrl_R01.fastq ctrl_R02.fastq --outSJfilterReads Unique --outSAMstrandField intronMotif --outFilterMultimapNmax 1
